# The Association Between Childhood Maltreatment and Somatic Symptoms in Adulthood

**DOI:** 10.1192/j.eurpsy.2025.166

**Published:** 2025-08-26

**Authors:** A. M. Luond, G. Ayas

**Affiliations:** 1 Adult Psychiatry and Psychotherapy, University Hospital of Psychiatry, Zurich, Switzerland; 2 Graduate School of Health Sciences, Koc University; 3 Affective Laboratory, Koç University Research Center for Translational Medicine (KUTTAM), Istanbul, Türkiye

## Abstract

**Abstract: Background:**

Childhood maltreatment (CM) encompasses various forms of abuse and neglect before age 18 and frequently manifests in somatic symptoms (SS) such as chronic pain or fatigue. Despite growing recognition of this connection, the relationship between specific CM types and SS, as well as the mechanisms underlying this link, remains incompletely understood.

**Objective:**

To examine the current understanding of the association between CM and SS, to highlight gaps in the literature, and propose directions for future research.

**Method:**

A state-of-the-art review searching a range of different databases was performed to explore the interplay between CM (exposure) and SS (outcome) in adults (over age 18).

**Results:**

Identified literature gaps include 1) inconsistency regarding the specific impact of subtypes of CM, specifically of neglect, on the development of SS; 2) narrowing the focus to specific functional syndromes (e.g., fibromyalgia), or selected health outcomes (e.g., respiratory disease) rather than SS as a broad category; and 3) underexploring the impact of culture.

**Discussion:**

Key recommendations for future research include adopting standardized WHO definitions for CM subtypes, expanding SS diagnostic criteria (e.g. through using comprehensive ICD-11 coding), and integrating cultural moderators (e.g. different health beliefs) into research methodologies. By adopting these recommendations, research could significantly improve patient care and mitigate the broader societal consequences of childhood trauma.

**Disclosure of Interest:**

None Declared

